# Tensile Deformation and Fracture Behavior of API-5L X70 Line Pipe Steel

**DOI:** 10.3390/ma15020501

**Published:** 2022-01-10

**Authors:** Mikhail L. Lobanov, Vladislav A. Khotinov, Vladimir N. Urtsev, Sergey V. Danilov, Nikolay V. Urtsev, Sergey I. Platov, Stepan I. Stepanov

**Affiliations:** 1 Heat Treatment & Physics of Metals Department, Ural Federal University, 19 Mira Str., 620002 Ekaterinburg, Russia; m.l.lobanov@urfu.ru (M.L.L.); v.a.khotinov@urfu.ru (V.A.K.); s.v.danilov@urfu.ru (S.V.D.); 2 M. N. Miheev Institute of Metal Physics, 18 S. Kovalevskaya Str., 620108 Ekaterinburg, Russia; 3 Ausferr Research and Technology Center, 11/1 Lomonosova Str., 455000 Magnitogorsk, Russia; urtsev@inbox.ru (V.N.U.); n.urtsev@ausferr.ru (N.V.U.); 4 Department of Metal Forming Machines and Technologies, Nosov Magnitogorsk State Technical University, 38 Lenina Ave., 455000 Magnitogorsk, Russia; psipsi@mail.ru

**Keywords:** TMCP, HSLA steel, deformation anisotropy, EBSD, Taylor factor, texture, necking ellipticity, splitting

## Abstract

Thermo-mechanical controlled processing (TMCP) is employed to obtain the required level of mechanical properties of contemporary HSLA steel plates utilized for gas and oil pipeline production. The strength and crack resistance of pipeline steels are mainly determined by its microstructure and crystallographic texture. In this study, the influence of the structural and textural states of industrially produced API-5L X70-X80 pipeline steels on tensile mechanical properties was analyzed. TMCP routes with different hot rolling temperatures and cooling rates were employed. The texture of steel was assessed using the Taylor factor, which was calculated based on electron backscatter diffraction (EBSD). The decrease in rolling temperature resulted in the sharper texture characterized by {001} planes banding (cleavage planes in the bcc lattice) parallel to rolling direction. The tensile deformation behavior at the stage of necking was determined by the crystallographic and morphological texture of the material and demonstrated significant anisotropy. Rupture of all investigated samples was accompanied by the development of splitting on the fracture surface. The splitting was localized in the rolling plane similar to the splitting in standard Charpy tests of pipeline steels.

## 1. Introduction

Quality of life improvement and intensive industrial manufacturing development require large amounts of energy carriers, such as hydrocarbons. Trunk pipelines are often employed to deliver hydrocarbons from distant reservoirs often distinguished by adverse climatic conditions [1,2]. One of the most promising ways to increase cost efficiency of trunk pipelines is to increase their operation pressure by using high-strength steel capable of operating in adverse climatic conditions [3,4,5,6,7,8]. High-strength pipelines have thinner walls; therefore, their utilization decreases metal consumption [4,9,10].

Contemporary low-carbon steels and a specific industrial method of their processing, that comprises controlled hot-rolling and adjustable accelerated cooling (thermo-mechanical controlled processing—TMCP), are used to obtain the required levels of mechanical properties in steel plates designed for natural gas and crude oil pipeline production [4,8,9,10,11,12,13,14,15,16]. Increased resistance to the localization of plastic deformation and failure resistance are characteristic features of pipeline products manufactured by means of TMCP.

Strength and crack resistance of pipeline steel are predominantly determined by its textural and structural state. A transition from ferrite–pearlite microstructures (average ferrite grain size ~5 µm) to structures with prevailing products of diffusionless transformation (primarily bainite; average grain size ~1 µm [6]) obtained by austenite overcooling by means of alloying and rapid cooling has allowed to carry out a transition from X52–X65 to X70–X80 and subsequently to X100 grades [11,15,16,17]. In [18], grain refinement is stated to be the only factor that ensures a simultaneous increase in strength and toughness of a material.

Running fracture formation poses the greatest threat in the operation of pipelines [19,20]. The state of plates for the production of pipelines plays a crucial role in pipeline resistance to so-called ductile running fracture. Enhancement of steel strength characteristics (for grades X70, X80 and higher) has had a considerably negative effect on the ability of semi-finished and finished pipeline products to arrest running ductile fracture [21,22,23].

Pipeline plates exhibit anisotropy of their mechanical properties due to their specific structural and textural states [5,21,22,23,24]. Texture can affect steel strength and crack resistance in different ways, depending on the direction. Both theoretical calculations and experimental data provide reliable evidence to conclude that the propagation of a brittle crack in α-Fe, i.e., in a metal material with BCC lattice, occur along {001} crystallographic planes [22,25,26,27]. Texture formation during TMCP was thoroughly studied in [23]. The results of these studies demonstrate that elongated structural fields with a texture containing a “dangerous” {001} plane parallel to an axis of a pipe are formed in pipeline steel after TMCP.

Pipeline steel impact toughness is commonly investigated by Charpy tests. However, the plane of a notch for standard Charpy specimens cut from pipeline semi-products do not coincide with the planes in which the major fraction of {001} lies. These planes are parallel to the direction of the main crack propagation in pipeline products. Therefore, impact toughness is not measured in the most brittle, i.e., most “dangerous” direction.

Splitting often occurs on the fracture surface of the Charpy specimens [21,22,28]. This splitting can be described as brittle cracks perpendicular to the main surface of the ductile fracture. They form in the vicinity of a propagating crack tip as a result of a triaxial stress state and due to the fact that under certain conditions a material can be ductile in one direction and brittle in another, thus exhibiting anisotropy of brittleness [23,24]. In [29], it was demonstrated that splitting does not have a significant influence on main crack propagation, i.e., they do not directly affect material fracture itself. However, the occurrence of splitting in a fracture surface serves as evidence of the abnormality of a material structure, since it indicates the presence of the relatively extensive fields with a homogenous texture, i.e., the regions where cracks can readily propagate in one direction (regions of the lowest fracture energy).

The advantage of the tensile testing in comparison with the other standard mechanical properties investigations is conditioned by the guaranteed specimen break. Moreover, contemporary methods of fracture initiation (necking) monitoring should provide valuable data on the characteristics of the ductile fracture of the analyzed textured materials (provided that the rolling direction and plane are precisely established).

This paper investigates the pipeline HSLA steel grade X70 plates produced at different TMCP controlled rolling temperatures and cooling rates. The crystallographic texture of the central regions of the pipeline steel plates after TMCP was evaluated. Based on EBSD data, the Taylor factor has been calculated for the studied specimens of pipeline semi-products in order to evaluate the effect of crystallographic texture on yield strength, uniform elongation and neck ellipticity, which were determined after tensile testing. Finally, we examine the effect of TMCP parameters on the features of resulting texture and the development of splitting during fracture of tensile specimens.

## 2. Materials and Methods

### 2.1. Material

**Figure 1:**
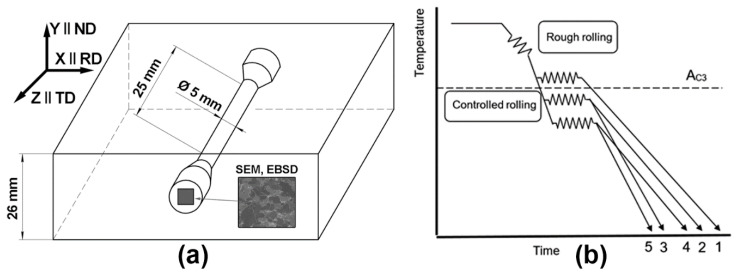
Schematic illustrations of cutting out of specimens for SEM and EBSD (**a**) and of the TMCP routes (**b**).

The study was conducted on specimens separated from the central regions of the plates (Figure 1a), which were obtained by pilot industrial TMCP (Figure 1b). HSLA steel, containing 0.05 wt. % C, 0.1 wt. % Si, 1.5 wt. % Mn, 0.2 wt. % Mo, 0.06 wt. % Nb, and 0.04 wt. % Al (the remainder comprising Fe and unavoidable impurities), was smelted in an oxygen converter and casted into 300 mm thick slabs, which were preheated at 1200 °C prior to TMCP. The total strain after rough rolling was 66.7%. The final rough rolling temperature measured on the surface of the plates was 970–990 °C. The finishing temperature of isothermal controlled rolling was controlled by the strain per pass, the number of passes and the dwell time between the passes. The total strain after controlled rolling was 86.7% and the finishing controlled rolling temperatures (T_CR_) at the last pass are shown in Table 1. The plates were cooled in a continuous controlled cooling unit. The cooling rates were controlled by the pressure and the flow rate of the water in a cooling unit. Table 1 shows the estimates of cooling rates for the central region of the plates in the temperature range from the T_CR_ to A_C1_ for all 5 processing routes. A_C3_ = 850 °C and A_C1_ = 670 °C were calculated using Thermo-Calc. The efficient non-recrystallization temperature T_NR_ = 976 °C was evaluated according to [30].

### 2.2. Specimen Preparation and Microstructure Orientation Analysis

A coordinate system (X, Y, Z) with X—parallel to the rolling direction (RD), Y—parallel to the normal direction (ND) and Z—parallel to the transverse direction (TD) was used in both the structural and textural analysis of the pipeline steel plates as shown in Figure 1a. The metallography was conducted on the samples separated 11–13 mm away from the surface of the plate. A scanning electron microscopy (SEM) investigation was conducted across the thickness of each specimen.

Sample surface preparation for electron backscatter diffraction (EBSD) analysis was carried out according to the method presented in [31]. Grinding was carried out on 3 types of sandpaper with particle sizes of 28–40, 18–14 and 10–14 µm for 15 min per sandpaper type. An electrolytic polishing of the samples in a solution of 15% HClO_4_ (perchloric acid), 85% CH_3_COOH (acetic acid) was performed at 21 V voltage after the grinding.

SEM was carried out on a Tescan Mira 3 (Tescan Orsay Holding, Brno—Kohoutovice, Czech Republic) with an auto-emission cathode at an accelerating voltage of 20 kV. An EBSD HKL Inca system with an Oxford Instruments analyzer (Carl Zeiss NTS, Oberkochen, Germany) was used for local texture analysis and to determine the orientations of individual grains (crystallites). The scanning step was 0.1 µm (100 nm). Orientation estimation inaccuracy did not exceed ±1° (±0.6° on average). The area under EBSD analysis was 100 × 100 μm^2^. Orientation distribution function (ODF) analysis and Taylor factor evaluation were performed for 4 neighboring areas of investigation in order to increase reliability of the data.

### 2.3. EBSD Study of the Average Grain Size

**Figure 2:**
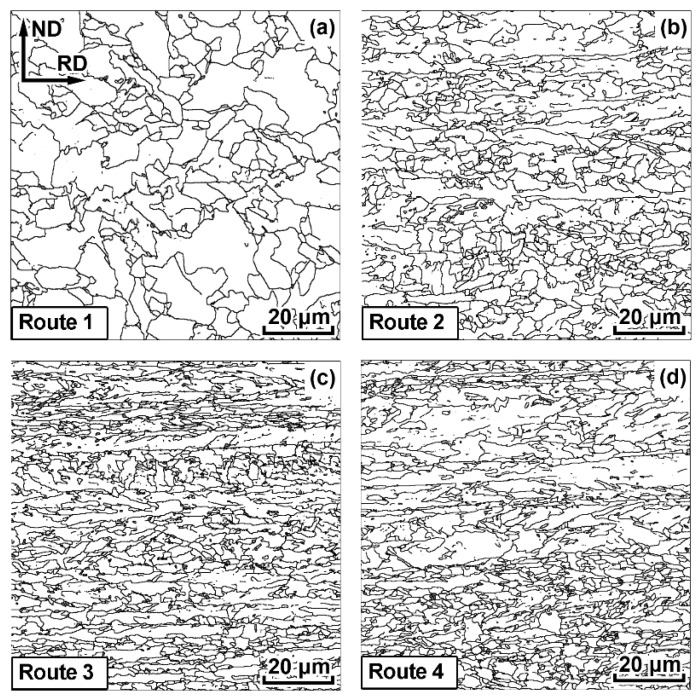
EBSD analysis of grain structure of the pipe steel specimens after TMCP.

The average size of all grains (crystallites) was determined in the microstructure of all specimens by means of EBSD, utilizing Oxford Instruments Channel 5 software. The average size was calculated as a diameter of a circle equivalent to an average area of crystallites. A crystallite was considered as an object circumscribed by boundaries with at least 7° misorientation angles. All of the crystallites, with the exception of the ones smaller than 1 µm, were analyzed (Figure 2).

### 2.4. Tensile Testing

Tensile testing ASTM E8/E8M-21 was carried out on standard cylindrical specimens (Figure 1a) with a diameter of 5 mm and a gauge length of 25 mm on an Instron 3382 testing machine (Instron, High Wycombe, UK) at a 5 mm/s test rate at room temperature. The specimen axis was parallel to RD, and the rolling plane was marked on a grip surface. Strain at the stage of necking was measured according to the method described in [32].

### 2.5. Taylor Factor Determination

Taylor factors were calculated for a uniaxial tension by means of Oxford Instruments software. Taylor factors serve as means to estimate yield stress of a polycrystalline sample, while taking into account the orientation hardness of each grain (σ**_y_** = Mτ*, where M—Taylor factor, τ*—critical resolved shear stress [33]). Taylor factors were calculated for all orientations identified on orientation maps and then averaged with the consideration of the orientation fraction.

## 3. Results

### 3.1. Microstructure

**Figure 3:**
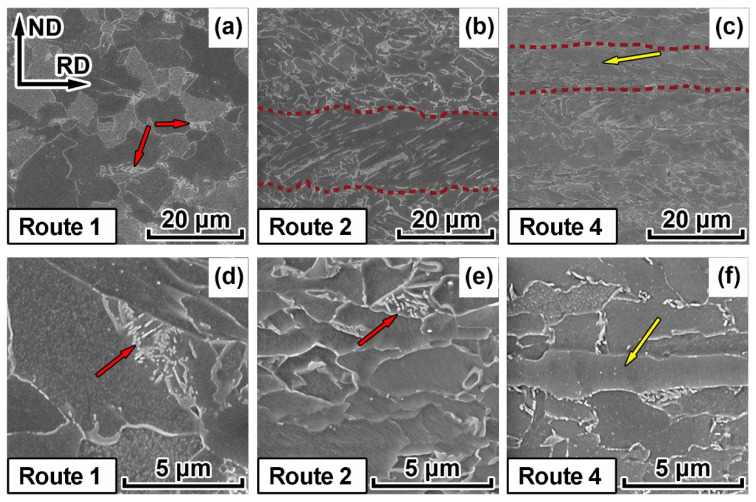
Scanning electron microscopy-backscattered electron (SEM-BSE) images of the central areas of the pipe steel plates after TMCP subjected to five routes: (**a**,**d**) route 1; (**b**,**e**) route 2; (**c**,**f**) route 4. Tempered martensite is marked with red arrows. Deformed ferrite grains are marked with yellow arrows. Red dotted lines indicate prior austenite grains.

SEM microstructure demonstrates areas elongated in RD (Figure 2 and Figure 3) for all TMCP routes with the exception of a high-temperature route 1. These up to 30 μm thick areas are significantly larger than the length of the investigated area and correspond to the prior austenite grains that have been elongated in RD during controlled rolling. They are characterized by a relatively homogenous internal microstructure and are indicated with red dotted lines in (Figure 3b,c). These crystallites typically represent displacive transformation products, in this case, bainite, according to [34]. Bainite crystallite size varies from 1 to 30 μm. Relatively small (1–5 μm) light areas marked with red arrows in Figure 3a,d,e can be observed on the bainite grain boundaries. These areas are distinguished by their own fine, often lamellar substructure consisting of several phases, which can be observed at higher magnification (Figure 3d,e). According to [35], these areas are classified as tempered lath martensite that were formed from carbon-rich austenite. In fact, tempered lath martensite contains extremely small and uniformly dispersed cementite particles embedded within a continuous ferrite lath.

Thin (less than 5 μm thick) regions elongated parallel to RD with poorly distinguishable internal structure can be observed for specimens 3, 4 and 5 (Figure 3c,f). Presumably, these areas correspond to ferrite, that has been formed during hot-rolling, i.e., has been deformed, recrystallized and preserved during cooling.

Decreasing the hot-rolling temperature to approximately Ac_3_ and/or increasing accelerated cooling intensity lead to a substantial refinement of the bainite microstructure (Table 1). In the case of the higher intensity cooling (route 3), fineness of the microstructure in the central areas of the plates increases by approximately 10 %. At the same time, decreasing hot-rolling temperature below Ac_3_ introduces ferrite grains elongated in the rolling direction (Figure 2b–d and Figure 3c,f), which complicates the integral estimation of the average grain size. Settings of the TMCP routes 2 and 3, as well as 4 and 5 contained similar controlled hot-rolling temperatures. The resulting shapes and sizes of crystallites obtained according to these routes were similar as well.

### 3.2. Crystallographic Texture

**Figure 4:**
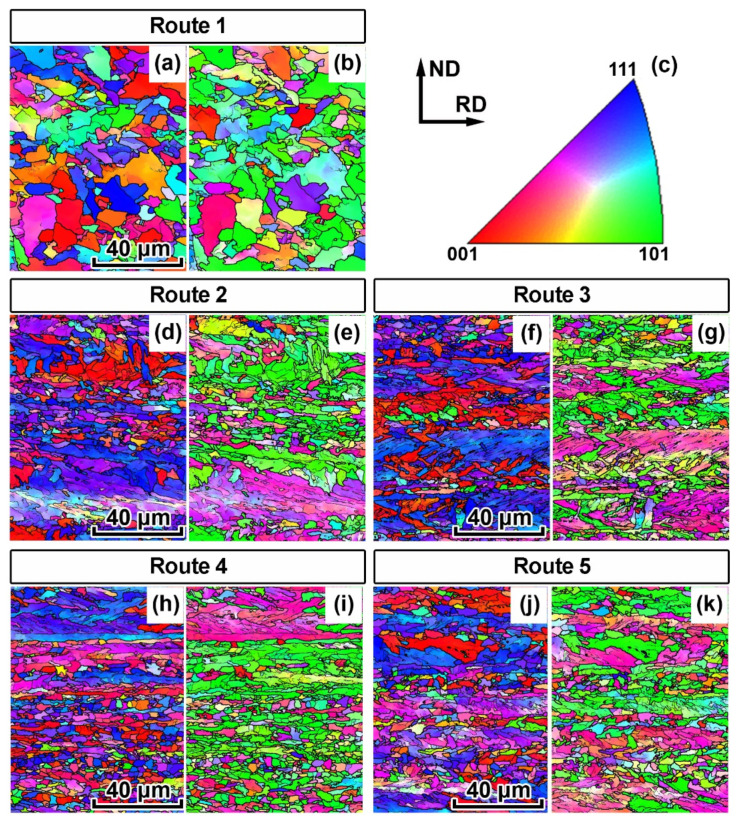
EBSD analysis of central regions of pipe steel plates obtained according to different TMCP routes: (**a**,**b**) route 1; (**d**,**e**) route 2; (**f**,**g**) route 3; (**h**,**i**) route 4; (**j**,**k**) route 5; (**c**) stereographic triangle with color-differentiated crystallographic directions; (**a**,**d**,**f**,**h**,**j**) orientation maps (coloring from ND||Y); (**b**,**d**,**f**,**h**,**j**) orientation maps (coloring from RD||X).

EBSD analysis reveals a pronounced crystallographic texture of all specimens that have been subjected to TMCP. Crystallographic directions <111> and <100>; <112> and <110> of most of the crystallites are parallel to ND and RD, respectively (Figure 4). This coincides with the TMCP texture presented in [21,36,37,38]. All preferred orientations of specimen 1 are significantly more scattered than those of other specimens. This is apparently due to ferrite recrystallization during controlled cooling from the finishing hot-rolling temperature.

**Figure 5:**
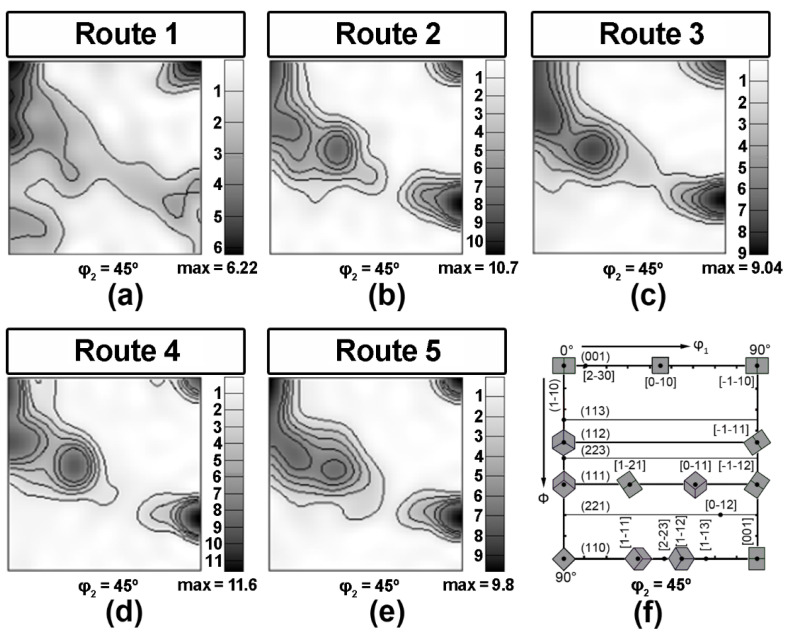
ODF cross-sections for φ_2_ = 45° from central area of pipeline steel plates after different TMCP routes (**a**–**e**), (**f**) standard φ_2_ = 45° ODF cross-section.

According to the analysis of ODF, the crystallographic texture of the plates subjected to all 5 TMCP routes is quite similar (Figure 5). Central area texture of all specimens determined by means of EBSD comprise a discrete set of scattered orientations (001)[1−10], (114)[1−10], (112)[1−10], (223)[2−52], (221)[−1−14]. Such texture is typical of HSLA steels after hot deformation [21,36,37,38] and develops due to the phase γ → α-transformation, which occurs according to the orientation relationship by Kurdyumov–Sachs (K-S) or Nishiyama–Wassermann (N-W). A discrete set of orientations obtained as a result of the multivariant phase transformation (1 → 24 for K-S and 1 → 12 for N-W) is explained in [21] by the nucleation of bainite at crystallographically ordered boundaries between austenite grains, which are characterized by the development of stable orientations of the FCC lattice after rolling.

For sample 1, all orientations are more scattered and two orientations (223)[2−52], (221)[−1−14] have significantly lower intensity compared with samples 2–5. This is apparently due to the recrystallization of α-phase resulted from the highest T_CR_ for Route 1. For samples 2–5, the orientations resulting from the γ → α transformation are observed alongside with the orientations typical of deformed and recrystallized ferrite grains, which according to [39] correspond to {hhl}<110>-type orientations. Such orientations of the α-phase develop during controlled rolling below A_C3_.

**Figure 6:**
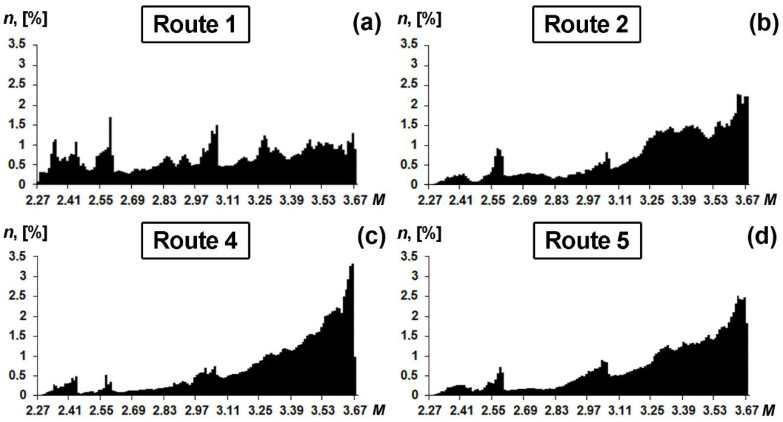
Taylor factors in TD for the central areas of the pipeline steel plates after TMCP.

The obtained values of the Taylor factor for a uniaxial stressed state are presented in Table 2. Specimens 2–5 have significantly different values of Taylor factor in all directions in comparison with those of specimen 1. Specimen 1 has a more uniform Taylor factor distribution, whereas in specimens 2–5 the center of gravity of orientation fraction shifts to the higher values of Taylor factor (Figure 6).

### 3.3. Tensile Testing

**Figure 7:**
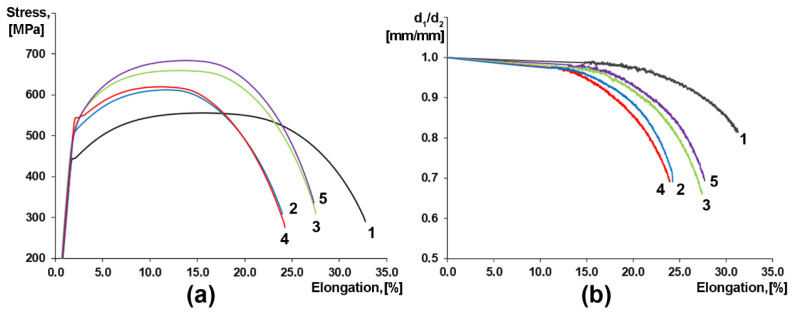
Stress–strain curves of the pipeline steel specimens after different TMCP routes (**a**) variation of elliptic semi-minor axis to semi-major axis ratio in specimen reduced cross-sections after tensile testing (**b**). Specimen axis was parallel to TD.

**Figure 8:**
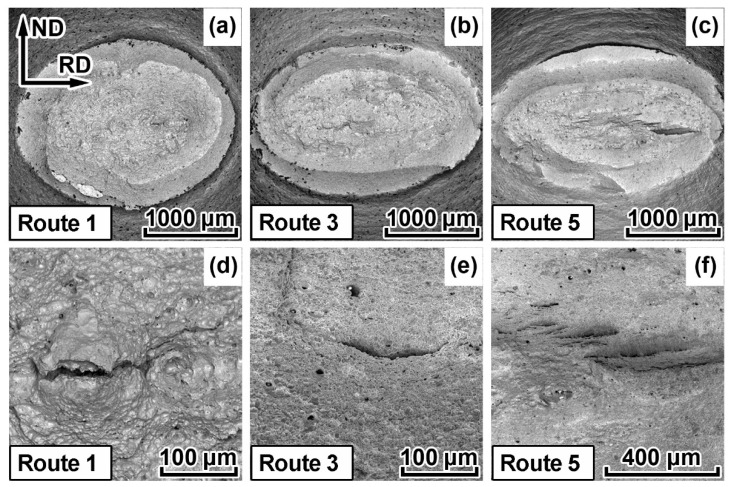
Fracture surfaces of cylindrical specimens of pipeline steel after standard tensile testing: (**a**,**d**) route 1; (**b**,**e**) route 3; (**c**,**f**) route 5.

Deformation behavior varies significantly depending on the TMCP route (Figure 7a), even though the fracture of all specimens occurred at almost identical stresses. Specimens 3 and 5, which have been cooled more intensively, simultaneously demonstrate higher uniform elongation and ultimate tensile strength in comparison with specimens 2 and 4, which have been cooled at lower rates after controlled rolling (Table 1). The cross-section of tensile specimens in the neck acquired an ellipsoid shape; major ellipse axes almost exactly coincided with RD and minor axes coincided with ND (Figure 7b and Figure 8a–c). Maximum ellipticity does not exceed 5% for specimen 4 at the uniform strain stage, while it can exceed 30% at the localized strain stage (necking), namely for specimen 5. Moreover, specimen 1, in which TMCP settings have included the highest finishing hot-rolling temperature, is distinguished by the minimal ellipticity.

Splitting appeared in the fracture surface of all the specimens despite the fact that fractures occurred at room temperature. Similar splitting often appeared in the fracture surfaces of the Charpy impact specimens of pipeline steels [21,27]. The plane in which the splitting occurred (especially in the central area of the reduced sections) is almost parallel to the TMCP rolling plane. Singular splitting was typical of specimens 1–4, while specimen 5 contained numerous separations (Figure 8c,f). It is also important to note that even though the nature of all the fractures is ductile, the surfaces of the metal at separation sites correspond to brittle fracture (Figure 8).

## 4. Discussion

Observed anisotropy of deformation and fracture during tensile testing results in the elliptical shape of the fracture area and in the development of splitting on the fracture surface of the specimens separated in the TD of the plate made of HSLA steel.

**Figure 9:**
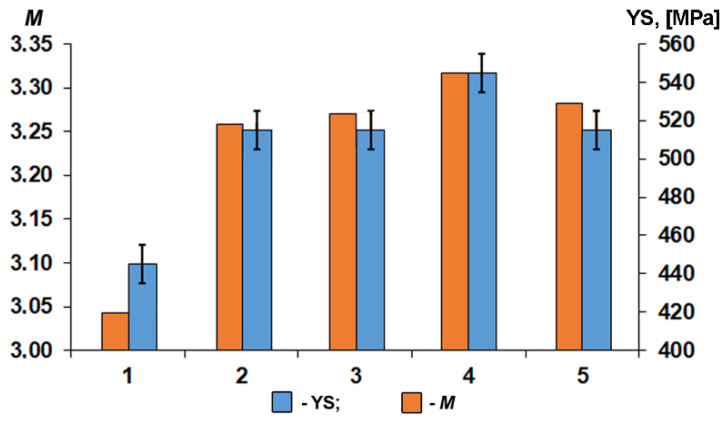
Comparison of yield stress with the averaged Taylor factors for pipeline steel specimens in TD.

We demonstrate the influence of crystallographic texture on deformation anisotropy by evaluating the integral Taylor factor of central area for HSLA steel processed with different TMPC parameters. The difference between Taylor factors demonstrates the difference between the resistances of crystallographic orientation to the initiation of plastic deformation. The analysis of Taylor factor distribution indicates a perceptible correlation with the yield stress determined by means of tensile testing (Figure 9). This confirms that the yield strength along TD is mainly attributed to the specimen texture state that has been developed during hot-rolling.

Tensile testing was carried out at samples separated parallel to TD. The texture in TD is described by the following set of orientations (001)[−1−10], (114)[−2−21], (112)[−1−11], (223)[−1−22], (221)[−1−10]. According to [40], the last three of the above orientations appear to be substantially harder in TD, i.e., a new coordinate system rotated at 90° about ND. In addition to a coarser grain size (Table 1), this explains the substantially lower yield stress for sample 1, for which the intensities of the orientations (223)[−1−22] and (221)[−1−10] were considerably weaker.

Uniaxial tension along the TD during mechanical testing should tend to the reorientation of grains and the development of texture with the <110> axis parallel to the direction of tension, i.e., TD in this case. The uniform elongation was equal to 9–12% for all studied samples. Such low strain at this stage of tensile testing should not result in significant changes in the texture according to [41]. However, at non-uniform stage of tensile testing the crystal lattice rotation transforms texture by dislocations gliding (in at least 5 independent slip systems) [42] in a crucially different way for the samples initially having different crystallographic and morphological textures.

**Figure 10:**
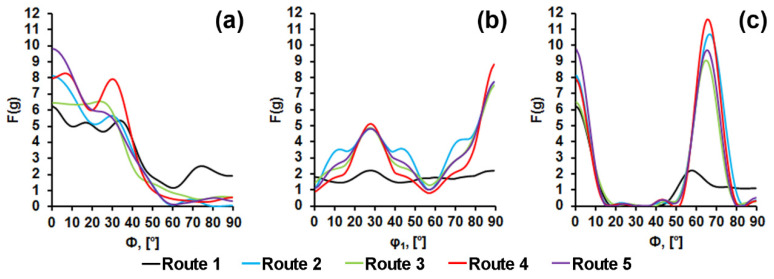
Normalized pole density distribution along Euler angles: (**a**) φ_2_ = 45°, Φ = 0–90°, φ_1_ = 0°; (**b**) φ_2_ = 45°, Φ = 55°, φ_1_ = 0–90°; (**c**) φ_2_ = 45°, Φ = 0–90°, φ_1_ = 90°.

**Figure 11:**
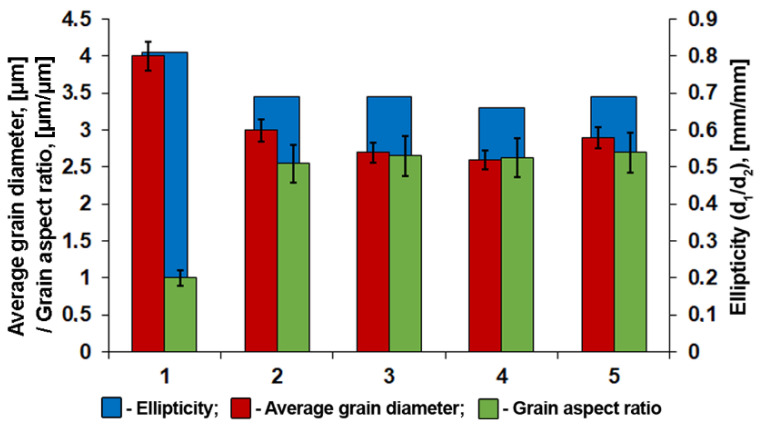
Dependence of tensile specimen fracture area ellipticity from crystallite shape in the central layers of pipeline steel plates.

A more isotropic state of sample 1 (Figure 5 and Figure 10) is due to the following factors: the scattering of preferred orientations, a relatively uniform distribution of the Taylor factor in TD (Figure 6) and a more equiaxial shape of the grains (Figure 11). These factors increase the uniform elongation, decrease the ultimate tensile strength and provide a rounder shaped residual fracture area in the tensile specimens for the TMPC route 1.

The combination of the hard (221)[−1−10] orientation in TD (Figure 10) with a finer grain size for samples 2–5, as well as an increased fraction of dispersed precipitates of tempered martensite, especially for which γ → α-the transformation took place at lower T_CR_ and higher cooling rates, is responsible for the high values of YS (Table 1). However, the specimens 2 and 4 with similar microstructure and texture were characterized by a 10% difference in YS. This might be due to strain ageing, which took place during controlled cooling.

The crystallographic texture, namely the pronounced {001}<110> orientation, which is soft in TD, RD and hard in ND, apparently results in a slightly higher ductility of samples 2–5 in TD (Figure 7a). This texture effect along with the grain aspect ratio results in a more elliptical shape of the fracture area in comparison with sample 1 (Figure 11).

The location of splitting on the fracture surface of tensile specimens remains constant for all specimens and TMCP routes; however, the nature of the splitting varies somewhat. The lower the rolling final temperature, the wider (the more grains are involved in) the separations (Figure 8). We have demonstrated that deformed ferrite grains elongated in RD are characterized by the {hhl} <110>-orientation. Moreover, the lower the T_CR_ the more fraction of such ferrite grains is observed. According to [23], the plane, in which splitting occurs during a standard Charpy test, corresponds to the {001}<110> orientation. In this study, we observed an increase in the intensity of this orientation for specimens 2–5 (Figure 5) with one of the {001} crystallographic planes parallel to the rolling plane and with one of the <110> crystallographic directions parallel to the RD.

## 5. Conclusions

1. The texture of the specimen central layers after all investigated TMCP routes was comprised of a number of scattered, and thus overlapping orientations: (001)[1−10], (114)[1−10], (112)[1−10], (223)[2−52], and (221)[−1−14]. Lowering the controlled rolling temperature and increasing the cooling rate were accompanied by a significant sharpening of the (223)[2−52], (221)[−1−14] orientations;
2. Orientation-averaged Taylor factor correlates with the plate yield stress measured in the transverse direction. The lowest YS was observed due to the greater grain size and increased fraction of soft {hhl}<110>-type orientations for the TMPC route with the highest finishing controlled rolling temperature above A_C3_;
3. Elongation to fracture of tensile specimens is determined by a combination of soft {hhl}<110> and hard (223)[2−52], (221)[−1−14] orientations relative to tension axis—TD. Ellipticity of the fracture area of tensile specimens decreases with a higher finishing rolling temperature and less pronounced texture;
4. The fracture of all studied specimens was accompanied by the development of splitting on the specimen fracture surface, similar to those that formed during the standard Charpy testing. More intense splitting was observed on the fracture surface of tensile specimens with a lower finishing controlled rolling temperature due to an increase in the fraction of ferrite deformed below A_C3_, which is characterized by {001}<110>-orientation.

## Figures and Tables

**Table 1:** Processing parameters, average grain size and tensile mechanical properties for five routes of TMCP.

Route	T_CR_/°C	V_CR_/°C/s	Average Grain/μm	YS/MPa	UTS/MPa	Non-Uniform Elongation/%	Total Elongation/%
1	920	25–45	4.0 ± 0.8	445 ± 15	560 ± 18	17.5 ± 1.0	30.0 ± 1.0
2	840	20–30	3.0 ± 0.4	515 ± 11	610 ± 12	14.0 ± 1.0	23.0 ± 1.0
3	840	25–35	2.7 ± 0.4	515 ± 9	660 ± 11	16.5 ± 0.7	27.0 ± 0.7
4	760	15–20	2.6 ± 0.4	545 ± 9	620 ± 10	15.0 ± 0.5	24.0 ± 0.5
5	770	20–30	2.9 ± 0.4	515 ± 7	685 ± 13	15.0 ± 0.6	26.0 ± 0.6

YS—yield strength, UTS—ultimate tensile strength, V_CR_—controlled cooling rate range, T_CR_—controlled rolling final temperature.

**Table 2:** Averaged Taylor factors of the central areas of the pipeline steel plates after TMCP.

Route	X (RD)	Y (ND)	Z (TD)
1	3.00	3.24	3.04
2	3.04	3.19	3.26
3	3.02	3.16	3.27
4	3.02	3.16	3.32
5	3.01	3.13	3.28

## Data Availability

The data presented in this study are available on request from the corresponding author.
